# A Chinese named entity recognition model incorporating recurrent cell and information state recursion

**DOI:** 10.1038/s41598-024-56166-3

**Published:** 2024-03-06

**Authors:** Qingbin Han, Jialin Ma

**Affiliations:** School of Computer and Software Engineering, Huaiyin Institute of Technology, Huaian, 223003 Jiangsu China

**Keywords:** Information technology, Computer science

## Abstract

Chinese is characterized by high syntactic complexity, chaotic annotation granularity, and slow convergence. Joint learning models can effectively improve the accuracy of Chinese Named Entity Recognition (NER), but they focus too much on local feature information and reduce the ability of long sequence feature extraction. To address the limitations of long sequence feature extraction ability, we propose a Chinese NER model called Incorporating Recurrent Cell and Information State Recursion (IRCSR-NER). The model integrates recurrent cells and information state recursion to improve the recognition ability of long entity boundaries. To solve the problem that Chinese and English have different focuses in syntactic analysis. We use the syntactic dependency approach to add lexical relationship information to sentences represented at the word level. The IRCSR-NER is applied to sequence feature extraction to improve the model efficiency and long-text feature extraction ability. The model captures contextual long-distance dependent information while focusing on local feature information. We evaluated our proposed model using four public datasets and compared it with other mainstream models. Experimental results demonstrate that our model outperforms traditional and mainstream models.

## Introduction

The NER is an essential and critical task in information extraction. Its objective is to extract, locate and classify entities with specific semantics from unstructured text^1^. This extracted information can be used in various natural language processing downstream tasks, such as information retrieval, knowledge graphs, and question-and-answer systems et al.^2–6^. Accurately identifying the attributes of named entities is valuable for knowledge representation and information extraction and is widely used.

The given text sequence is often labelled in NER using sequence annotation methods. There are three common annotation methods for entity identification sequences, namely Begin Inside Outside (BIO), Begin Middle End Single (BMES), and Begin Inside Outside Single End (BIOSE) annotation schemes. Reimers et al.^7^. compared IOB, BIO, and BIOES labelling schemes. Their experiments demonstrate that the BIO and BIOES annotation schemes are superior to the IOB scheme in the NER task. Therefore, our experiment used BIO labelling methods.

Chinese NER techniques have evolved from traditional approaches to deep learning. Early methods based on rules and dictionaries^8^ required the creation of specific rule templates. As this method depends on creating knowledge bases, it often results in low portability. NER methods based on statistical machine learning relied on selecting and analyzing features, which consumed a lot of manpower. However, deep neural networks with powerful feature learning capabilities have been applied to NER in recent years, achieving excellent results. Hammerton^9^ was the first to combine Long Short-Term Memory (LSTM) with CRF for NER. To address the problem of not being able to obtain contextual bi-directional sequence information, Lample et al.^10^. proposed Bi-directional Long Short-Term Memory (Bi-LSTM) combined with the CRF network model. However, the capture of long-distance dependence caused information loss.

To address the limitations in extracting features from long sequences, this study introduces a Chinese named entity recognition (NER) model called Incorporating Recurrent Cell and Information State Recursion (IRCSR-NER). By incorporating recurrent cells and information state recursion, the model enhances its ability to accurately identify long entity boundaries. Additionally, to address the syntactic differences between Chinese and English, we introduce dependency-based syntactic analysis at the input representation layer of Chinese NER. Specifically, we employ a syntactic dependency approach to incorporate lexical relationship information at the word level in sentence representations. This approach is applied in conjunction with IRCSR-NER for sequence feature extraction, resulting in improved efficiency and long-text feature extraction capability. The model effectively captures contextual long-distance dependencies while simultaneously focusing on local feature information. Experimental results demonstrate that the proposed model achieves the best performance in terms of the F1 value on four public datasets when compared to other mainstream models. Overall, this method significantly enhances the model's ability to accurately identify long entities in lengthy texts and exhibits generalization capabilities for Chinese entity recognition tasks.

The main contributions of this paper are as follows: (1) We propose a model that combines recurrent cancer cells and information state recursion (IRCSR) for named entity recognition (NER). By applying IRCSR-NER at the sequence encoding layer, the model captures long-distance contextual dependencies while focusing on local feature information. (2) Our work demonstrates that enhancing the recognition of entity boundaries and improving the performance of Chinese NER can be achieved by incorporating part-of-speech relationship information into Chinese sentences. (3) Through experiments conducted on four public datasets, we demonstrate that our IRCSR-NER model achieves better results in terms of F1 score compared to traditional models and existing state-of-the-art models. We further validate the effectiveness of the syntactic dependency module, the information state recursion module, and the overall IRCSR-NER structure through ablation experiments.

The paper is structured as follows: Section "Related research" provides an overview of related research and background knowledge. In Section "Methods", we present a detailed description of the proposed IRCSR-NER model and its core components. Section "Experimentation and analysis of results" focuses on the experimental design and provides details about the datasets used. In this section, we also present the experimental results with analysis and discussion. Finally, in Section "Conclusions", we summarize the paper and discuss future research directions.

## Related research

The sequence encoding layer is crucial in deep learning models for named entity recognition (NER). It is responsible for encoding the input sequence into semantic representations, which are then used to annotate the sequence at the output layer. In the past, recurrent neural networks (RNNs) were primarily used for sequence encoding, but they had limited capabilities in modeling long-range dependencies^11^. Recently, the development of self-attention mechanisms has led to the emergence of fully attention-based networks, such as Transformers, which have replaced RNNs as the mainstream approach. Transformers have shown more effectiveness in capturing global dependencies. Notably, inter-layer self-attention in SPAN-BERT^12^ and bidirectional transformers in OntoNLP^13^ have shown significant improvements in feature extraction capabilities. When performing entity extraction tasks on knowledge graphs, the incorporation of different lexical information has been widely applied. For example, the introduction of rule-based lexical information has been extensively utilized^14^.

For rule-based entity tasks, the part-of-speech (POS) information among words in a sentence is a crucial component. Dependency parsing, also known as dependency analysis, is a commonly used method in relation extraction. It involves analyzing the grammatical structure of a sentence to identify relevant words and their relationship types. Tian et al.^15^ further improved the predictive accuracy by combining dependency parsing with the bidirectional affine attention mechanism. Fei et al.^16^ addressed the issue of overlapping entity relation extraction and proposed a method that treats the overlapping relation extraction task as a quintuple prediction problem. This method achieved advanced performance by modeling entity relation graphs using graph attention models. In order to leverage the syntactic knowledge effectively in information extraction tasks, Fei et al.^17^ proposed a novel structure-aware GLM model. This model greatly addresses the issue of long-range dependencies and boundary recognition, while emphasizing the effectiveness of syntactic structural information in information extraction tasks. Therefore, this paper chooses to incorporate syntactic dependency methods to add POS relationship information to the entity recognition task.

The CNNs are commonly used in the sequence encoding layer of Chinese NER models. Gui et al.^18^ introduced a parallel sentence matching approach that combines a dictionary with a CNN-based reflection mechanism. Shi et al.^19^ developed the CNN-Head Transformer Encoder (CHTE) model to handle multi-word expressions, but it comes with increased computational costs. It utilizes CNNs with varying window sizes to capture attention head vectors, thereby enhancing local features while preserving global semantics. In order to simultaneously address flat, overlapping, and disjoint entity recognition, Li et al.^20^ proposed a model that models the adjacent relationships between entities. This model represents unified NER as a two-dimensional network of word pairs, incorporating multi-granularity 2D convolutions to optimize grid representations.

The RNNs incorporate "recurrence" to retain previous states and impact subsequent outputs, capturing contextual information effectively. However, when dealing with long sequences, RNNs are susceptible to the problems of vanishing or exploding gradients^21^. Dong et al.^22^ employed bidirectional LSTM-CRF for Chinese NER, leveraging both character-level and partial-level representations. This study was the first to investigate partial-level performance within this architecture for Chinese NER, achieving state-of-the-art results at the time.

The Transformers are also employed as sequence encoding layers, relying solely on attention mechanisms without the use of recurrence or convolution. They offer reduced training time and demonstrate remarkable performance in addressing natural language problems. Yan et al.^11^ presented a NER architecture that incorporates a flexible Transformer Encoder for character and word representations. Li et al.^23^ proposed the plane lattice deformation model, which takes advantage of the robust capabilities of Transformers and meticulously crafted position encodings to fully exploit lattice information, transforming the lattice structure into spans.

Extensive research has been conducted to address the challenges of entity recognition by incorporating different lexical information. Fei et al.^24^ explored the task of biomedical information extraction (BioIE). To tackle the issue of existing information extraction methods neglecting the fusion of external structured knowledge, they proposed a method that integrates large-scale biomedical knowledge graphs to enrich contextualized language models. To address the problem of overlooked discontinuous and irregular entity recognition, Fei et al.^25^ introduced a NER model called BIOBERT + MAPtr, which utilizes a pointer network. The model's pointers contain positional information that represents entity labels. Zhang et al.^26^ proposed the Lattice LSTM model, which incorporates lexical information into LSTM. However, Lattice LSTM is not parallelizable and struggles to handle lexical conflicts effectively. Sui et al.^27^ presented the Collaborative Graph Network (CGN) model, which combines dictionary and character information at the encoding layer and employs graph attention networks for encoding. This model also resolves the issues of Lattice LSTM by including self-matching word information and closest-word information. Nonetheless, differences still exist between the results obtained from graph structures and sequence structures due to their inherent disparities.

While sequence encoding methods have shown promising results in NER, they still have some limitations in modeling tasks. The CNN can only capture local features and falls short in modeling long-distance dependencies. The RNN encounters issues of vanishing and exploding gradients when dealing with lengthy sequential text, and it has slower training speed. Although Transformers theoretically have the ability to model dependencies across any distance, the practical application of dependency modeling is still limited. To address these challenges, we propose a novel structure called Integrated Recurrent Cell and Information State Recursion (IRCSR-NER) as a sequence encoding layer. In the input representation layer of Chinese NER, dependency syntactic analysis is introduced. The model combines recurrent cells with information state recursion. We integrate this structure with pre-trained language models and Conditional Random Fields (CRF) to form a joint learning model. By utilizing recurrent units and information state recursion, the model enhances the recognition capability of long entity boundaries. Applying IRCSR-NER to sequence feature extraction improves the efficiency and effectiveness of extracting features from lengthy texts. The model's input layer incorporates lexical relation information into the sentence representation at the word level, thus enhancing the entity boundary recognition capability. The model captures both long-distance contextual dependencies and local feature information. Experimental results demonstrate that this approach enhances the model's ability to accurately identify long entities in long texts.

## Methods

**Figure.1:**
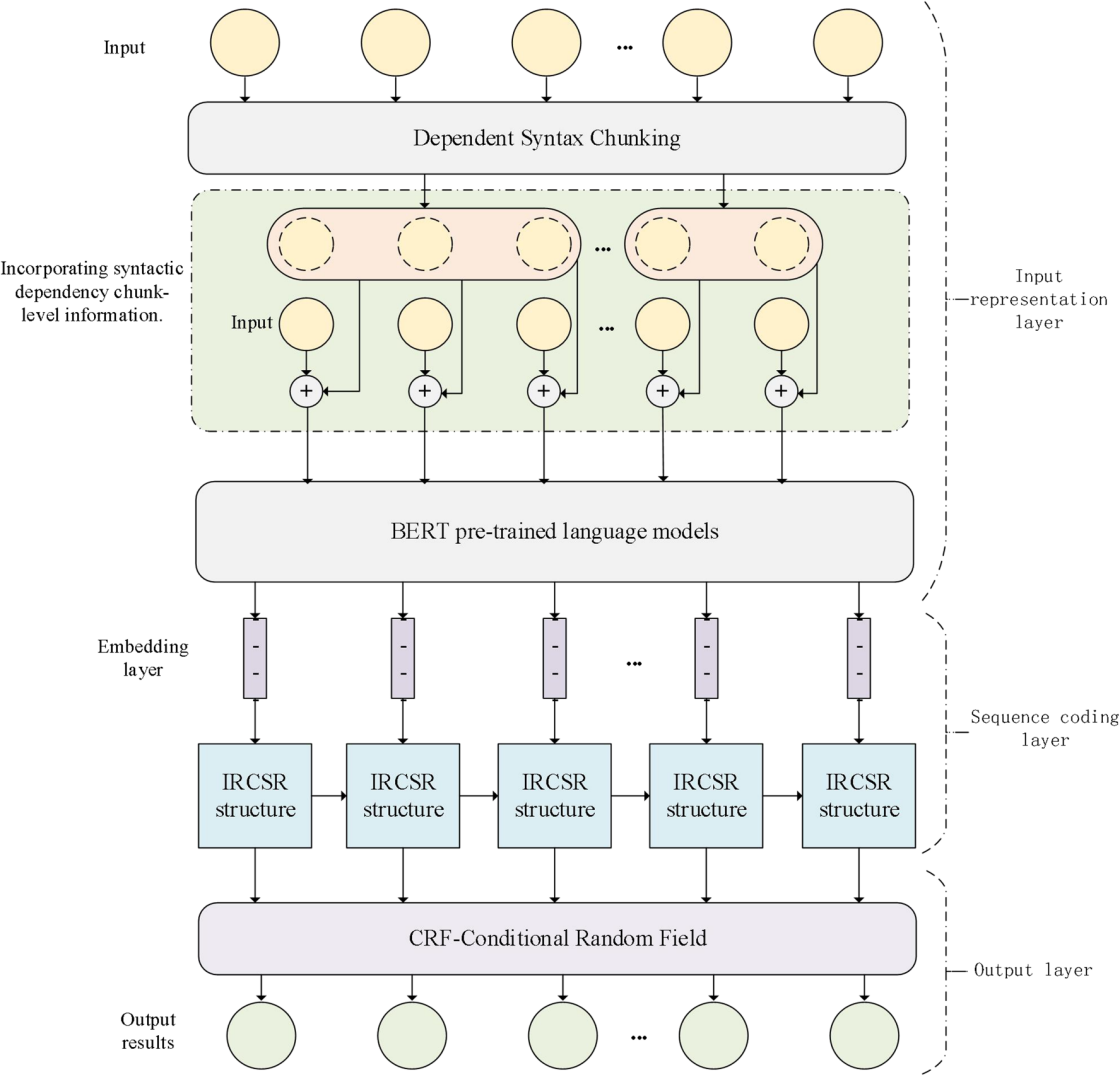
Application of IRCSR-NER's entity recognition model.

Our proposed IRCSR-NER structure serves as a sequence coding framework within a joint learning model. Our proposed NER model comprises three layers: an input representation layer, a sequence encoding layer, and an output decoding layer, as shown in Fig. 1. We introduce dependent syntactic analysis to the input representation layer specifically for Chinese NER. Dependent syntactic analysis is a technique that aims to uncover the semantic structure of a sentence by examining the dependencies between its components. In contrast to English, where syntactic analysis relies on spatial relationships between sentence components, Chinese syntactic analysis focuses on the dependency relationships between these components. Our model is inspired by Hutchins et al.^28^ who applied recurrent units to classification tasks. In our approach, the input representation layer gathers block-level information from syntactic dependency chunking. This information, which reflects rich semantic knowledge, is leveraged for downstream sequential coding tasks through pre-training language models with BERT. The sequence encoding layer incorporates multiple IRCSR structures. The input sequence is horizontally extracted using the Transformer with Attention mechanism to extract features. The state information from the previous moment is interactively updated with semantic vectors, while the new semantic vector is passed to the subsequent moment. Vertically, the Transformer with attention mechanism efficiently computes Self-Attention and Cross-Attention for feature extraction. Finally, the sequence encoding information is outputted to the decoding layer, which predicts the corresponding out sequence text.

### Syntactic dependency chunking

In this paper, we introduce dependent syntactic analysis to the input representation layer of Chinese NER. The goal of dependent syntactic analysis in Chinese is to reveal the semantic structure of a sentence through the dependencies between its components. Unlike English syntactic analysis, which is based on the concept of space, Chinese syntactic analysis is based on the dependency relationship between sentence components. We use the HanLP Chinese natural language processing tool to implement neural network-based Chinese dependency syntactic analysis. Based on lexical relations, we perform syntactically dependent chunking of the input sequence text. The chunking information is then assembled into the original sequence text.

**Figure.2:**
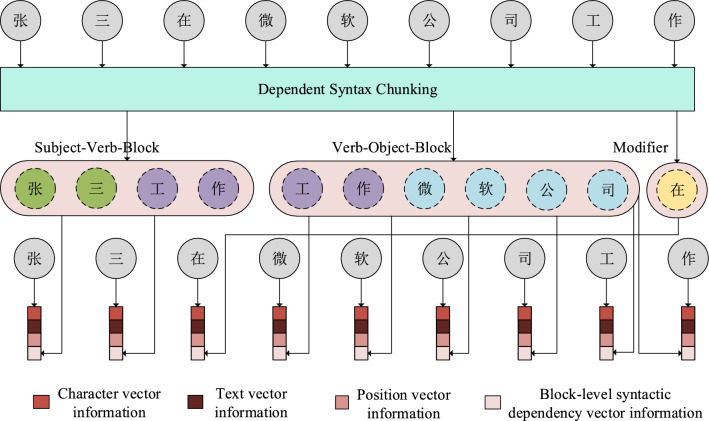
Example results of syntactic dependency blocking information.

Based on the Dependency Syntax Tree, a sentence can be divided into several semantically meaningful chunks. Each of the chunks contains a central word and its closely related constituents. We break down sentences into separate components (such as subject-verb and verb-object blocks, as well as modifier blocks) based on their respective subtrees in the tree structure. These blocks are then assembled into a vector representation of the input sequence, as shown in Fig. 2. This facilitates the downstream sequence annotation task to focus on semantic information.

In Fig. 2, we can see that the subject "张三" and the predicate "工作" are part of the Subject-Verb-Block. The predicate "工作" and the object "微软公司" are part of the Verb-Object-Block. The remaining modifying relations, adverbs, prepositions, auxiliaries, and other lexical words are also part of the Verb-Object-Block. The predicate "工作" and the object "微软公司" are also part of the Verb-Object-Block. The rest of the modifier relations, prepositions, and other lexical words such as "在" are part of the individual Modifier-Block. We add the information of the constituent block containing each word in the input vector information. This distinguishes the core constituents, reduces the interference between them and helps downstream models focus on the entity itself. Words in the constituent block have a close local dependency and carry rich local semantic information, which is useful for knowledge expression.

### Incorporating recurrent cell and information state recursion (IRCSR-NER)

**Figure.3:**
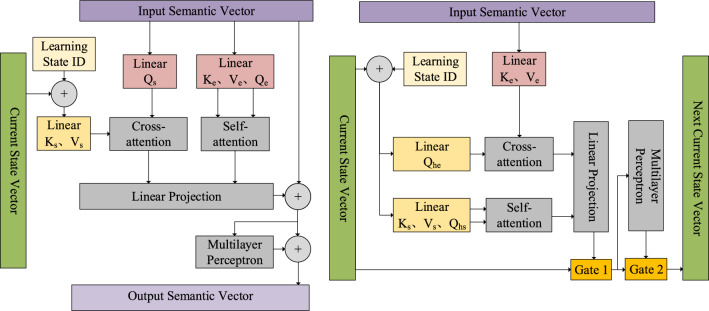
The structure of IRCSR-NER vertical and horizontal.

**Figure.4:**
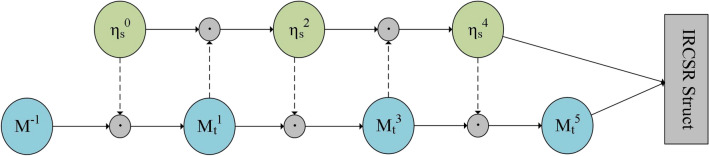
The structure of IRCSR-NER status update.

The IRCSR units receive two inputs: a set of semantic vectors from the input representation layer and a set of current state information vectors. The IRCSR operation consists of three components: the vertical direction, the horizontal direction, and the state information recursion. The process of transforming input semantic vectors into output semantic vectors in the decoding layer is referred to as the vertical direction. The process of transitioning from the current state vector to the next state vector is referred to as the horizontal direction. The recursive operation of propagating information in the horizontal direction is known as state information recursion. Figure 3 and Fig. 4 illustrates this process.

According to Fig. 3, the purpose of the vertically oriented Transformer unit is to obtain the feature representation. It performs self-attention computation on word sequences and cross-attention computation on word sequences and state vectors. The self-attention and cross-attention mechanisms compute the vector values of the query (query vector), value (information vector), and key (key vector). The inputs of *Linear*(*K*_*s*_*,V*_*s*_) and *Linear*(*Q*_*s*_) are the word sequence vector *M*_*t*_ and the state vector *C*_*l*_ incorporating the learning state, respectively. Additionally, The input of *Linear*(*K*_*e*_*,Q*_*e*_*,V*_*e*_) is the word sequence vector *M*_*t*_, and the state vector *C*_*l*_ is denoted as:

1 $$C_{l} = \eta_{s}^{ - 1} + L_{id}$$

Equation (1) introduces the input state vector at the previous time step, denoted as *η*_*−*_1* s*, and the learned state ID, *L*_*id*_, which is employed to label the IRCSR structural unit.

To compute the self-attention representation *s*_*a*_ and the cross-attention representation *c*_*a*_, distinct vector values are employed. Specifically, for the self-attention representation, the vector values *Q*_*e,p*_, *V*_*e,p*_, and *K*_*e,p*_ are obtained from the query vector, value vector, and key vector, respectively, using the *Linear*(*K*_*e*_*,Q*_*e*_*,V*_*e*_) operation. These values are utilized to determine the self-attention weights. On the other hand, for the cross-attention representation, the vector values *Q*_*s,p*_, *V*_*s,p*_ and *K*_*s,p*_ are obtained from the query vector, value vector, and key vector, respectively, by applying the *Linear*(*K*_*s*_*,V*_*s*_) and *Linear*(*Q*_*s*_) operations. These values are employed to calculate the cross-attention weight. The computation process is illustrated in Eqs. (2) and (3).

2 $${\text{s}}_{{\text{a}}} = soft\max \left( {\frac{{Q_{e,p} \times K_{e,p}^{T} }}{{\sqrt {{\text{d}}_{k} } }}} \right) \times V_{e,p}$$

3 $${\text{c}}_{{\text{a}}} = soft\max \left( {\frac{{Q_{s,p} \times K_{s,p}^{T} }}{{\sqrt {{\text{d}}_{k} } }}} \right) \times V_{s,p}$$

To derive the vertical word sequence feature vector, a series of computations is employed. Initially, the self-attention and cross-attention operations are linearly projected, yielding a linear projection vector, *l*_*p*_. Subsequently, the word sequence vector, *M*_*t*_, and the linear projection vector, *l*_*p*_, are processed through a vertical feedforward fully connected network junction output, denoted as *FFV*(*M*_*t*_ + *l*_*p*_). Finally, the word sequence vector, *M*_*t*_, the linear projection vector, *l*_*p*_, and the feedforward fully connected network junction output, *FFV*(*M*_*t*_ + *l*_*p*_), are fused to generate the vertical word sequence feature vector, *M*_*t,n*_. The computation process is illustrated in Eqs. (4) and (5).

4 $${\text{l}}_{p} = W_{{{\text{\_1}}}} \times s_{a} + W_{{{\text{\_2}}}} \times c_{a}$$

5 $$M_{{\text{t,n}}} = FFV(M_{t} + l_{p} ) + l_{p} + M_{t}$$

In the equation, *W*_*_*1_ and *W*_*_*2_ represent two linear transformation matrices. The variables *s*_*a*_ and *c*_*a*_ correspond to the self-attention representation and the cross-attention representation, respectively, computed in Eqs. (2) and (3).As illustrated in the horizontal structure depicted in Fig. 3, the loop unit performs self-attention computations on the input word sequence. Additionally, the loop unit conducts cross-attention computations on the word sequence vectors and state vectors. The gating unit is utilized to implement the forgetting mechanism, which controls the flow of information computed by the attention mechanism. The structure also generates a new state vector through interaction with the state vector to update the sequence vector from the previous time step. This new state vector is then propagated to the next time step. The query vector, value vector, and key vector values are computed using both the self-attention mechanism and the cross-attention mechanism. The inputs for *Linear*(*K*_*s*_*,V*_*s*_) are the word sequence vectors, *M*_*t*_. The inputs for *Linear*(*K*_*s*_*,Q*_*hs*_*,V*_*s*_) and *Linear*(*Q*_*he*_) are the state vectors of the fused learning state. The state vector, *C*_*l*_, is defined in Eq. (1).

During the calculation of the self-attention representation, the vector values *Q*_*hs,t*_, *V*_*s,t*_ and *K*_*s,t*_ derived from the query vector, value vector, and key vector using *Linear*(*K*_*s*_*,Q*_*hs*_*,V*_*s*_) are employed to compute the self-attention weights. In the computation of the cross-attention representation, the cross-attention weights are computed using the vector values *Q*_*he,t*_, *V*_*e,t*_ and *K*_*e,t*_ obtained from the query, value, and key vectors through *Linear*(*K*_*e*_*,V*_*e*_) and *Linear*(*Q*_*he*_). The calculation process is illustrated in Eqs. (6) and (7).

6 $$s_{{{\text{ha}}}} = soft\max \left( {\frac{{Q_{he,t} \times K_{e,t}^{T} }}{{\sqrt {{\text{d}}_{k} } }}} \right) \times V_{e,t}$$

7 $${\text{c}}_{{{\text{ha}}}} = soft\max \left( {\frac{{Q_{hs,t} \times K_{s,t}^{T} }}{{\sqrt {{\text{d}}_{k} } }}} \right) \times V_{s,t}$$

Linear projection is applied to the self-attention and cross-attention to obtain the linear projection vector, *l*_*hp*_, as calculated in Eq. (8):

8 $${\text{l}}_{hp} = W_{{{\text{\_3}}}} \times s_{ha} + W_{{{\text{\_4}}}} \times c_{ha}$$

In the equation, *W*_*_*3_ and *W*_*_*4_ represent two linear transformation matrices. The variables *s*_*ha*_ and *c*_*ha*_ correspond to the self-attention representation and the cross-attention representation, respectively, computed in Eqs. (6) and (7).

The output *f*_1_ of the word sequence vector, *M*_*t*_, and the linear projection vector, *l*_*hp*_, after undergoing the gating unit is calculated as demonstrated in Eq. (9).

9 $${\text{f}}_{1} = \sigma \left( {W_{{{\text{\_5}}}} \times l_{hp} + W_{{{\text{\_6}}}} \times \eta_{s}^{{_{{^{ - 1} }} }} } \right)$$

In the equation, *W*_*_*5_ and *W*_*_*6_ represent two linear transformation matrices and *σ* is the sigmoid activation function.

To compute the horizontal state vector,* η*_*f*_, which represents the output of gated unit 2, the output *f*_1_ of gated unit 1 is combined with the output of the feedforward fully connected network junction. This fusion is accomplished using gating unit 2, yielding the desired horizontal state vector.

The calculation process for deriving the horizontal state vector through gating unit 2, as illustrated in Eq. (10), is as:

10 $$\eta_{{\text{f}}} = \sigma \left( {W_{{{\text{\_7}}}} \times FFV(f_{1} ) + W_{{{\text{\_8}}}} \times f_{1} } \right)$$

In the equation, *W*_*_*5_ and *W*_*_*6_ represent two linear transformation matrices, *η*_*−*_1* s* is the input state vector at the previous moment, and *σ* is the sigmoid activation function.

In the IRCSR model illustrated in Fig. 3, the keys and values (*K*, *V*) are shared between the vertical and horizontal segments. We calculate one set of keys and values (*K*_*e*_*, V*_*e*_) from the input semantic vectors, and another set (*K*_*s*_*, V*_*s*_) from the state vectors. Each segment has its own unique query vector (*Q*), leading to four different sets: *Q*_*e*_ and *Q*_*s*_ for the vertical segment, and *Q*_*he*_ and *Q*_*hs*_ for the horizontal segment. The query vectors *Q*_*e*_, *Q*_*s*_, *Q*_*he*_, and *Q*_*hs*_ are four distinct and non-overlapping vectors. This approach is designed to enhance the model's versatility and separability. By incorporating multiple relevant features within the sequence, it enables the model to improve its expressive power and generalization ability.

As shown in Fig. 4, we input the acquired state vector and the word sequence vector from the previous moment into the recursive update module. This module determines the number of rounds to operate on the horizontally propagated state vectors by judging whether it's an odd or even number of rounds. For odd rounds, we use Eq. (11), and for even rounds, we use Eq. (12). Finally, the state vector *η*_*f*_ is obtained after an odd number of rounds into the next loop cell.

11 $$\eta_{s} = 2\sigma \left( {Q_{i} \times \eta_{prev}^{i - 1} } \right) \odot {\text{M}}_{t - 1}^{i - 2}$$

12 $$\eta_{{_{prev} }}^{i} = 2\sigma \left( {R_{i} \times {\text{M}}_{t - 1}^{i - 1} } \right) \odot \eta_{{_{prev} }}^{i - 2}$$

where σ is the sigmoid activation function, *Q*_*i*_ is the random initialisation matrix,* M*_*t−*1_ is the block of semantic vectors input at the previous moment, and *η*_*prev*_ denotes the state vector computed at even numbers.

### CRF-conditional random field

Conditional Random Field (CRF) is used to globally predict the labelled sequence of any given sequence. Given an input sequence *X* = {*x*_1_*,x*_2_*,…,x*_*n*_} and a labelled sequence *Y* = {*y*_1_*,y*_2_*,…,y*_*n*_} can be computed *s*(*X,Y*) as:

13 $$s(X,Y) = \sum\limits_{i = 0}^{{\text{n}}} {A_{i,j} } + \sum\limits_{i = 1}^{n} {P_{i,j} }$$

where *A* is the transfer matrix. *P*_n*k_ is the label score of the encoder and *k* is the number of label types. *A*_*i,j*_ represents the score from label *i* to label *j*.

The probability of optimizing the label sequence:

14 $$p(Y|X) = \frac{{e^{s(X,Y)} }}{{\sum\limits_{{y \in Y_{X} }} {e^{{s(X,\widehat{{\text{y}}})}} } }}$$

where *Y*_*X*_ represents the set of all possible labelled sequences.

In the training phase, the maximum logarithmic probability of correct prediction is considered. For decoding, the labelled sequence that has the highest score for the input sequence is filtered as the final output:

15 $$y^{*} = \mathop {\arg \max s}\limits_{{\widehat{y} \in Y_{X} }} \left( {X,\widehat{y}} \right)$$

## Experimentation and analysis of results

### Datasets

The MSRA dataset is a Chinese named entity recognition dataset provided by Microsoft Research Asia, specifically in the news domain. It consists of over 50,000 Chinese sentences with entity annotations. The entities are categorized into three types: Person, Location, and Organization.The Resume dataset is generated by filtering and manually annotating the summary data of senior executives' resumes from listed companies on the Sina Finance website. It includes 1027 resume summaries, and the entities are classified into eight categories: Person, Country, Location, Race, Profession, Education, Organization, and Title. The OneNote dataset is derived from a large manually annotated corpus. It includes 18 entity types, including Person, Location, and Organization.The Weibo dataset is generated by filtering historical data from Sina Weibo between November 2013 and December 2014. It contains 1890 Weibo messages, and the entities are classified into four categories: Person, Location, Organization, and GPE (Geopolitical Entity).

### Experiments configuration

All our experiments were conducted on an NVIDIA Quadro RTX4000 GPU for training purposes. The model parameters are presented in Table 1. During the training process, we utilized a Chinese pre-trained language model, BERT, for pre-training. The model employed the Adam optimization function, with the numerical stability parameter epsilon set to 1e−8. The learning rate was set to 3e−5, and the hidden layers had a dimensionality of 768. We employed the IRCSR-NER architecture for sequence encoding tasks, where the number of multi-head attention heads was set to 8, and each head had a dimensionality of 64. The model utilized hidden layers with a dimensionality of 768, an encoding depth of 6, a feed-forward layer expansion ratio of 4, and 4 recursive iterations. During the information state recursion, we employed 5 rounds of information interaction. We applied dropout with a probability of 0.5 at all layers. The maximum sentence length in this study was set to 256 tokens, and the batch size was set to 16. We present the most optimal outcomes for all metrics obtained in the experiment.

**Table 1:** Model parameter description.

Parameter	Value
Optimizer	Adam
Epsilon	1e−8
Learning rate	3e−5
Hidden layer dimension	768
Dropout	0.5
Max sentence length	256
Batch size	16
Depth of encode	6
Dimension of attention head	64
Number of multi-head attention	8
Feed forward expansion ratio	4
Layers of recurrent	4
Interaction round	5

### Evaluation indicators

The following three mainstream evaluation metrics are mainly used in Chinese NER experiments: Precision (P), Recall (R), and F1-Score (F1). The specific calculations of these three evaluation metrics are shown in Eqs. (16–18):

16 $$P = \frac{TP}{{TP + FP}}$$

17 $$R = \frac{TP}{{TP + FN}}$$

18 $$F_{1} = 2 \times \frac{P \times R}{{P + R}}$$

The TP represents the number of positive classes that are correctly predicted to be positive. FN represents the number of positive classes that are predicted to be negative. The FP represents the number of negative classes that are predicted to be positive. The TN represents the number of negative classes that are correctly predicted to be negative.

### Analysis of results

In this paper, we propose a Chinese named entity recognition model called IRCSR-NER, which incorporates syntactic dependency information in the input representation and combines recurrent units with information state recursion in the sequence encoding process. In this section, we analyze and discuss the performance of different models on four public datasets. We compare the performance of three different types of entity recognition models: adaptive base framework, graph structure, and adaptive embedding. We present the optimal results for all metrics in the experiments.

Table 2 shows the experimental results on the Resume dataset and MSRA dataset. On the Resume dataset, the BERT-IRCSR-CRF model achieves the highest precision, recall, and F1 score. Compared to the BERT-PLTE-BIGRU-CRF^29^ model that incorporates positional information and a porous mechanism, our model improves precision, recall, and F1 score by 0.26%, 1.01%, and 0.63%, respectively. This demonstrates the effectiveness of our model in identifying multiple labels and capturing long-distance dependency information. On the MSRA dataset, our model achieves the highest recall result. The precision and F1 score obtain the second-highest results, surpassing the adaptive embedding model MECT^30^ and the graph structure model FGN^31^. This indicates that our model exhibits comprehensiveness in the task of entity capturing with standardized data.

**Table 2:** Results obtained on Resume and MSRA.

Mould	Resume			MSRA		
	P	R	F1	P	R	F1
BERT-LRCNN	94.50	92.93	93.71	96.29	94.97	95.63
Lattice-LSTM	94..81	94.11	94.46	93.57	92.79	93.18
BERT-BILSTM-CRF	95.75	95.28	95.51	95.06	94.61	94.83
TFM-BILSTM-CRF	96.37	96.01	96.19	95.37	94.84	95.11
BERT-PLTE-BIGRU-CRF	96.16	96.75	96.45	**96.47**	96.69	**96.58**
LGN	95.28	95.46	95.37	94.19	92.73	93.46
FGN	95.57	95.46	95.51	94.91	94.15	94.53
WC-GCN	95.05	94.82	94.94	95.53	92.42	92.97
MECT	96.40	95.39	95.89	94.55	94.09	94.32
Visphone	96.09	96.44	96.26	96.31	95.83	96.07
BERT-IRCSR-CRF	**96.42**	**97.76**	**97.08**	95.83	**96.84**	96.34

Significant values are in bold.

Table 3 presents the experimental results on the Weibo dataset and OneNotes dataset. On the Weibo dataset, due to the nature of short text messages in Weibo, our model achieves the second-highest accuracy and recall. However, our model obtains the highest F1 score, surpassing the Visphone^32^ model, which ranks second in terms of effectiveness, by 0.06%. This demonstrates that our model has the ability to capture both long entity boundaries and focus on short entity boundaries. On the OneNotes dataset, our model achieves the best results in terms of accuracy and F1 score. This indicates that our model is applicable to handling complex contextual relationships and complex entity boundaries.

**Table 3:** Results obtained on Weibo and OneNote.

Mould	Weibo			OneNote		
	P	R	F1	P	R	F1
BERT-LRCNN	57.14	66.67	59.92	76.40	72.60	74.45
Lattice-LSTM-CRF	53.04	62.25	58.79	76.35	71.56	73.88
BERT-BILSTM-CRF	69.65	64.62	67.33	81.99	81.65	81.82
BERT-PLTE-BIGRU-CRF	**72.00**	66.67	69.23	79.62	81.82	80.60
LGN	55.34	64.98	60.21	76.13	73.68	74.89
FGN	69.21	70.60	69.90	81.23	83.26	82.19
WC-GCN	55.38	62.98	59.31	75.05	72.29	73.64
MECT	61.91	62.51	63.30	77.57	76.27	76.92
Visphone	65.65	**71.29**	70.79	80.57	**84.79**	82.63
BERT-IRCSR-CRF	70.53	71.17	**70.85**	**82.16**	83.51	**82.83**

Significant values are in bold.

To validate the effectiveness of the IRCSR structure, ablation experiments were conducted. We primarily employed three ablation conditions: -Syntactic Dependency Information (SDI), -Information State Recursion Module (SR), and -IRCSR structure. -SDI denotes the removal of syntactic dependency information from the input representation layer. -SR signifies the elimination of the information State recursion module within the IRCSR structure. -IRCSR indicates the removal of the entire IRCSR structure.

According to the statistical analysis, when using models without the SDI module for entity recognition, the F1 scores decreased by 0.49%, 0.52%, and 1.19% on the Resume dataset, MSRA dataset, and OneNote dataset, respectively. On the Weibo dataset, the accuracy and F1 scores decreased by 0.27% and 0.03%, while interestingly, the recall rate increased by 0.21%. This can be attributed to the interference of syntactic dependency methods in handling unstructured texts, such as the Weibo dataset, leading to mislabeling some entities as negative examples. When the SR module was removed for entity recognition, the F1 score on the Weibo dataset showed the largest decrease of 2.57% among all datasets. This is because the State Representation module has a more comprehensive ability to capture crucial contextual information. For texts with high importance on contextual information, the SR module can better capture long-distance dependencies within the sequence. When the IRCSR module was removed from the model for entity recognition, the F1 scores decreased by 1.4% and 2.58% on the Resume dataset and MSRA dataset, respectively. On the Weibo dataset and OneNote dataset, the F1 scores decreased by 7.05% and 4.9%, respectively.

Table 4 presents the varying degrees of F1 score reduction across the four datasets when removing the SDI module, SR module, or IRCSR module. Analyzing the changes in accuracy, recall, and F1 score during the ablation experiments leads to the following conclusions:(1) The syntactic dependency method incorporates the grammatical relationships between words into the model, enabling better handling of data sparsity and improved contextual support. However, challenges still exist when dealing with informal and unstructured texts. (2) The utilization of horizontal recursive information propagation in the IRCSR model enhances the model's ability to handle complex sentences. This model improves the limitations of information propagation, particularly in long sequences. (3) By employing the IRCSR-NER structure as the entity recognition sequence encoding layer, the model effectively focuses on local feature information while capturing long-distance contextual dependencies. This demonstrates the effectiveness and robustness of the IRCSR-NER structure.

**Table 4:** Ablation experiments results on Resume, MSRA, Weibo and OneNote dataset.

Dataset		BERT-IRCSR-CRF	-SDI	-SR	-IRCSR
Resume	P(%)	96.42	95.86	95.63	94.87
	R(%)	97.76	97.34	97.06	96.50
	F1(%)	97.08	96.59	96.34	95.68
MSRA	P(%)	95.83	95.49	94.97	93.40
	R(%)	96.84	96.15	95.74	94.12
	F1(%)	96.34	95.82	95.36	93.76
Weibo	P(%)	70.53	70.26	68.35	65.77
	R(%)	71.17	71.38	68.21	62.05
	F1(%)	70.85	70.82	68.28	63.80
OneNote	P(%)	82.16	80.88	79.63	76.01
	R(%)	83.51	82.40	81.72	79.96
	F1(%)	82.83	81.64	80.66	77.93

**Figure.5:**
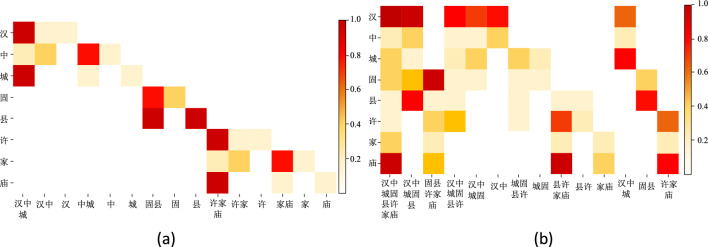
Example of character weight heat map: (**a**) BERT-CRF, (**b**) BERT-IRCSR-CRF.

The Chinese NER is a complex process where one character can correspond to multiple words. To observe the performance of the BERT-CRF model and the BERT-IRCSR-CRF model, we recorded their character weights for the sentence "汉中城固县许家村" and drew a heat map (Fig. 5). By comparing the results, we noticed that the BERT-CRF model pays more attention to the characters "城", "县", and "庙" when identifying the address entity "汉中城固县许家村". However, this model incorrectly identifies the sentence as three address entities—"汉中城", "固县", and "许家村". After consulting our entity thesaurus, we discovered that most entities have secondary and tertiary structures. In such cases, the entity boundary recognition ability plays a vital role in identifying named entities.

Our IRCSR-NER model can reduce the recognition ability of first-level and second-level endings while strengthening the recognition ability of lower-level endings. The heat map shows that "汉" and "村" carry the highest weight. The result indicates that the IRCSR structure can effectively improve entity boundary recognition for medium and long entities while reducing the interference of irrelevant endings.

To evaluate the effectiveness of the IRCSR structure in enhancing the performance of the Chinese NER model. We conducted an analysis of the difference in entity detection results based on several examples.

**Figure.6:**
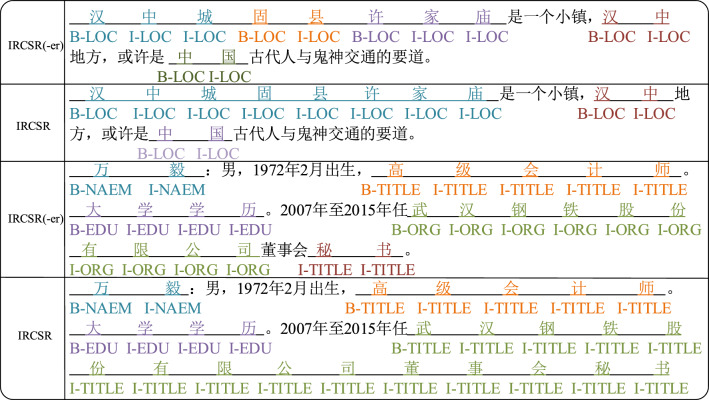
Example analysis and comparison.

Figure 6 clearly illustrates that the general network structure IRCSR(-er) model, which doesn't use the IRCSR structure in the first sentence, can accurately detect the place names (LOC) "汉中" and "中国". However, it incorrectly identifies "汉中城", "固县" and "许家庙" in "汉中城固县许家庙" as three separate LOCs. In contrast, the IRCSR model can correctly identify "汉中城固县许家庙" as a single place name (LOC). Similarly, in the second sentence, the IRCSR(-er) model correctly detects the name (NAME) "万毅", occupation (TITLE) "高级会计师", and education (EDU) "大学学历". However, it incorrectly identifies "武汉钢铁股份有限公司董事会秘书." as an organization name (ORG) and occupation (TITLE). On the other hand, the IRCSR model can correctly identify "武汉钢铁股份有限公司董事会秘书" as an occupation (TITLE). The above experimental results fully demonstrate the effectiveness of the IRCSR-NER model. It can improve the problems of incorrect detection of long entity nouns and insufficient ability to capture long-distance dependent information.

## Conclusions

In this work, we propose a Chinese NER model named IRCSR-NER. To enhance the identification of entity boundaries, we incorporate word information into the word-level representation of sentences via a syntactic dependency approach at the input layer of the model. We employ the IRCSR structure to sequence feature extraction, which improves its efficiency and ability to extract features from long texts. The model is designed to capture contextual long-range dependent information, while also focusing on local feature information. Compared to the five mainstream models, it achieved the highest accuracy, recall, and F1 values on the Resume dataset and the highest recall on the MASR dataset. There are many directions for our future work. One of the promising directions is to introduce multimodal network architecture in our model. Using correlated images to better recognize named entities with polysemous words contained in the text.

## Data Availability

The datasets used and analysed in the current study are available from the corresponding author upon reasonable request.
